# Reasons for low level of skilled birth attendance in Afar pastoralist community, North East Ethiopia: a qualitative exploration

**DOI:** 10.11604/pamj.2018.30.51.14420

**Published:** 2018-05-21

**Authors:** Mohammed Ahmed Ibrhim, Meaza Demissie, Araya Abrha Medhanyie, Alemayehu Worku, Yemane Berhane

**Affiliations:** 1 School of Public Health, Samara University, Samara, Ethiopia; 2 Addis Continental Institute of Public Health, Addis Ababa, Ethiopia; 3 School of Public Health, Mekelle University, Mekelle, Ethiopia; 4 School of Public Health, Addis Ababa University, Addis Ababa, Ethiopia

**Keywords:** Pastoralist, health facility delivery, home delivery, Ethiopia, Afa

## Abstract

**Introduction:**

Ethiopia has expanded the number of health facilities that offer maternal health services during the last two decades. However, the utilization of skilled birth attendants in health facilities is still very low especially among the pastoralist regions of the country. This study explored why women in the pastoralist region of Afar, Ethiopia still prefer to give birth at home.

**Methods:**

A qualitative study approach was used to collect information from October to December 2015. A total of eighteen focus group discussions and twenty-four key informant interviews were conducted. Focus group discussions were separately conducted with mothers and male tribal or religious leaders. Key informant interviews were conducted with heads of Women's Affairs Bureau, district health office heads and traditional birth attendants. Data were coded and categorized using open code software for qualitative data management and analyzed based on a thematic approach.

**Results:**

Women preferred to deliver at home due to lack of awareness about the benefits of maternity health facilities, their nomadic lifestyle, lack of confidence and trust in health workers and their close affinity and easy access to traditional birth attendants. Supply-side barriers included distant health facilities, lack of transportation and poor health care.

**Conclusion:**

Demand and supply related factors were identified as barriers to utilization of skilled birth attendants. Increasing awareness, bringing the service closer, arranging maternity waiting area around health facilities, and creating client-friendly service were found critical. Future research to define and improve services and approaches suitable for pastoralist population is warranted.

## Introduction

Pregnancy and childbirth-related complications are highly prevalent among women in low-income countries [1]. Although effective interventions to reduce maternal complications and improve survival of both the mother and offspring are available still, the utilization has been very low in many low-income countries [2-4]. Ethiopia is one of the countries with the highest maternal mortality ratio in sub-Saharan Africa, 412/100,000 live births [5]. In Ethiopia efforts to increase utilization of skill attendants at birth has been intensified in the last two decades by building new health facilities, deploying trained health workers, offering free delivery services and providing transportation (ambulances) for referrals [6,7]. These efforts have increased the utilization of skilled birth attendants from 18 to 60% in the country as a whole. However, no remarkable improvement has been recorded among the pastoralist regions of this country [5,6,8]. The factors associated with variations in maternal health services utilization across the regions in Ethiopia are multifaceted encompassing both supply and demand domains. Meager transport infrastructure, poor quality of services, prevailing traditional/cultural practices and low decision making power of women are among the common contributing factors [8-11]. However, lack of competent local researchers has hampered the conduct of research in the Afar region of Ethiopia. Thus, very little is known about the reasons that specifically hinder utilization of skilled birth attendants in the vast region of Afar. Understanding the maternal health services utilization patterns and the barriers of the Afar pastoralist women can generate useful information to improve maternal health services for the pastoralist communities particularly in this country and the continent at large. Therefore, this study was conducted to explore reasons for continued preference to giving birth at home despite the remarkable improvements made inaccessibility of health facilities in the pastoralist communities in the Afar region of Ethiopia.

## Methods

### Study area

The study was conducted in the six districts of Afar regional state of Ethiopia where more than 86% of the population lives in rural areas and about third fourth are pastoralists. The majority of Afar (Danakil) population is Muslims. Afar has the lowest altitude in Africa and hot dry climatic conditions that force pastoralist communities move constantly in search of grazing land and water. Ethiopia has a three-tier health system consisting of primary health care units which include health centers with satellite health posts at the base, district hospitals in the middle, and specialized hospitals at the top tier. According to Afar regional health bureau report, the region has six hospitals, 86 health centers and 379 health posts. In the study districts, three hospitals and 14 health centers with 69 satellite health posts were found functional. All hospitals and health centers provide basic obstetrical services. The health posts do not provide delivery services. Each district has an ambulance stationed at the district health office to facilitate referral cases whenever necessary and the ambulance driver has a mobile phone for communication.

### Study population and sampling

In the study area, the participants were selected from different population groups. The participants for the focus group discussions (FGD) were mothers who had children less than 24 months of age, grandmothers and recognized male tribal or religious leaders. All were selected purposively mainly based on either their experience of birth and birth-related traditions or being as influential persons. A total of 186 individuals participated in 18 FGDs; 60 mothers and 48 grandmothers and 54 male tribal or religious leaders. Key informant interview (KII) participants consisted of heads of district health office, women's affairs office and traditional birth attendants who were providing delivery services in the community. Key informant interviews (KII) were carried out with a total of 24 participants; six district health office heads, six women's affairs office heads, and 12 traditional birth attendants (TBAs). TBAs were selected in consultation with health extension workers (HEW), health workers, and the women's association office. The main criteria for their selection was being an active service provider and knowledgeable about the local culture.

### Data collection

Data were collected from October to December 2015. Semi-structured and open-ended FGD and interview guides were developed to guide the data collection. The main focus was on the reasons for choosing home delivery. Six midwives fluent in the local language (Afar) conducted the FGD sessions and key informant interviews after receiving a thorough training. The training provided by the principal investigator was focused on facilitation and interviewing techniques. FGDs and interviews were conducted in the Afar language and were closely supervised by the principal investigator. FGDs took up to two hours while key informant interviews took up to 60 minutes. The FGDs were conducted by two midwives; one serving as moderator and the other as note taker. Informed oral consent was obtained from all participants. All FGDs and KI interviews were recorded using a digital recorder. Audio records were transcribed verbatim and field notes were later integrated into the transcript.

### Data analysis

The transcribed data were loaded on to the open code software developed by Umea University in Sweden for coding qualitative data and assist in analysis [12]. The transcripts were read repeatedly, coded and organized into categories. Similar categories were grouped into themes: reasons for preferential utilization of home delivery, and health facility-related factors. Findings are presented in narratives with supporting quotations.

### Ethical approval

Ethical approval for the study was obtained from Mekelle University. Permission to conduct the study in the region was obtained from Afar Regional Health Bureau and from local administrative and health authorities. Verbal informed consent was obtained from each participant. Privacy and confidentiality of the participants was maintained by conducting FGDs and interviews in private places where intrusion by others was controlled. The anonymity of the data were assured by not documenting the full name of participants.

## Results

All women participants in the study were housewives with pastoralist lifestyle. Most of the women participants were married (82.4%), and others either divorced or widows. All participants were muslims and most (89%) of them were unable to read and write. Majority of the women, 47 of the 60, gave birth at home assisted by traditional birth attendants; nine delivered at health facilities due to birth complications and four women had normal delivery at a health facility. Among the 24 key informants selected from district offices, 6 were males and 18 females. Each TBAs had at least six years of experience in providing delivery services, however, all of them were unable to read and write and were muslim.

### Reasons related to planning delivery

**Lack of knowledge and information:** The majority of the women had no -knowledge about availability and benefits of delivery facilities. The majority of mothers considered childbirth as a natural process that should take place at home following the local customs and traditions and only in case of any complication to be referred to a health facility. The Traditional Birth Attendants are often the ones advising the family to take a laboring mother to a health facility when the delivery is not going well.

"There is no problem with home delivery! My mother gave birth to all five of her children at home with no problem and I so far delivered four at home safely. My mother is a TBA and she can manage any complication during labor except when the mother needs fluid and injection to stop bleeding". (FGD mother participant, 35years)

It was also noted that delivery by a skilled birth attendant in a health facility does not necessarily avoid unwanted pregnancy outcomes. Mothers believe that maternal deaths don´t always occur at home, can occur in health facilities as well.

"What does a mother get by delivering at health facilities? We witnessed the death of a mother who was referred by a TBA to a health facility, she died there and her body came back on the second day but her child was alive…"(FGD mother participants, 38 years)

Key informants from the district health bureau and women's affair bureau also recognized that adequate information is not available about the delivery options to help women in the community to make choices.

"Women in rural areas lack information about the advantage of using health facilities for delivery, pregnancy care and other maternal health services. Two health extension workers per/kebele are not enough to reach the widely scattered households in this pastoralists community" (KII participant district health bureau head, 45 years).

**Pastoralist way of life and its challenges:** The health facilities are not well organized to serve the mobile communities who live scattered in the vast drylands of the Rift Valley. A typical pastoralist woman has no permanent residence and is moving from place to place to find grazing land and water as a part of everyday life which may affect her pregnancy and childbirth outcomes. Their husbands may not be around when labor starts and may be far from health facilities compelling them to use of delivery facilities according to the preferences of the population.

"A woman told her own story…" it was the last month of my pregnancy and I was out in the field…I felt wet between my legs and wanted to go back home but I could not because labor pain started immediately. I stayed under a tree, luckily, there was a woman who could call a TBA for me and this TBA assisted the delivery under the tree" (FGD women participants, 29 years).

On the other hand, during adverse conditions such as drought, there is a great loss of cattle and other perishable resources. Under such circumstances, all male community members move out far in search of new places for grazing, often leaving their women somewhere safe. Pregnant women in labor in such a situation have no option but to give birth to their children wherever they are. One woman told her story as follows:

"Sometimes back, my husband went in search of water and grass for our cattle, he left me (I was pregnant) and my children in a house of TBA from his tribe. I experienced a serious bleeding during that birth…it was very difficult to reach to the nearest health facility…there were no men around to carry me and no ambulance, later somehow people who were around managed to take me to the nearest health facility and I was saved." (FGD Woman participants,21 years old).

**Economical reasons (poverty) :** Financial constraints are among the key factors that prevent pastoralist mothers from seeking delivery care at health facilities. The majority of participants (both male and female) described poverty as the main contributing factor. Although in actual practice delivery services are free of charges in public health facilities in Ethiopia, families may not afford to buy drugs and pay for transportation and other travel-related expenses. The majority of the women mentioned that in case of emergencies ambulance service is not easily available and accessible to them.

"In most of the cases, the reason why we don't seek service from skilled birth attendants is that we lack money at hand, even if we wish to go to a health facility, we can't afford all the costs related to travel and food… Drugs are often not available in health facilities and need to be purchased from private pharmacies at an exaggerated price. Health facilities are far from where we live, therefore we need money for transportation…"(FGD male participants,58 years).

" Transportation cost is expensive, it could be more to going back home after delivery, and we cannot afford that, therefore we prefer to stay home". (FGD women participants,32 years).

Another woman said

"Usually, pastoralist women don't have money and their husbands do not save money for such cases, they have to sell an animal when something happens…same for hospital expenses…so there is no readily available cash to pay for emergency events (KIIs District head,45 years old).

## Health services related-factors

**Distance and transport availability:** Most of the respondents mentioned that distance up to health facilities and inadequate transport service to reach a health facility is a major obstacle to utilize delivery service. Moreover, the remote nature and the dry hot weather conditions in the region make walking difficult and discourage women from seeking skilled delivery services.

"The area is remote and arid, there is no road access to most places in the district, therefore, there is no transportation service. These days there is an ambulance serving the whole district, often it's not accessible…it provides limited services" (KIls Male,59 years)"

"It is not easy to carry a pregnant woman on a locally made stretcher and then walk for hours. That is why delivery takes place at home in most of the times. (KIIs Women´s affairs bureau head,37years)

"Home is preferred for delivery due to the hot temperature that doesn't allow pregnant women to reach the health center on feet, in fact, walking on the hot sandy soil for a long time could be very dangerous and risky particularly to the pregnant woman. So women prefer to give birth at home ´´ (FGD health office head 55 years)

"During my first pregnancy, I was examined in a health facility, after investigation the health worker told me that I must give birth at a health facility under supervision of a skilled person. When the time came I wanted to go but couldn't because my village is too far from the health center, the weather was too hot, it takes at least an hour to walk to where I can get a bus to take me to a health facility. Thus, I was forced to give birth at home". (FGD Women participants,23 years old)

"I had a long labor, I was taken to a health center by ambulance, after examination, the midwife told me that my labor has not yet started…and there was no way to go back home, no transportation options. My mother asked the health worker if there is any waiting area or bed until labor starts, he (the midwife) showed her an open space in front of the delivery room… after a day of waiting there labor started. Imagine, how can you go and come back to health facilities in such circumstance?" (FGD women participants,20 years).

**Lack of confidence in health workers:** The unfriendliness of some health workers discourages pregnant women from seeking care at the health facilities. Participants also mentioned that some health workers lack proper skill and confidence to handle delivery.

"My daughter who was in labor pain went to a health center; a young nurse examined her immediately and referred her to a nearby hospital…she was not able to handle normal delivery…shortly after we left the health center, on the way to the hospital, my daughter gave birth in an ambulance and the delivery was handled by a TBA who was accompanying us. (KIIs TBA participant, 65 years).

Another reason for avoiding institutional delivery is the desire to re-suture part of the vagina after delivery. Traditionally Afar woman suture part of the vagina after delivery. This is something which can't be done at health facility so they prefer to give birth at home. One of the participants expressed this in the following manner.

"After giving birth women want the suture back, however health workers refuse to suture the parts in the genitalia that were sutured before. In my case, the health worker said that is not medically appropriate. However, after I returned home my mother corrected it, so why should I use health facility if they are not respecting my interest" (FGD Women participants 20 years old).

**Quality of health services:** Participants also complained about the lack of equipment, supplies, and drugs necessary for maternity care in health facilities. This shortage of materials also contributes partly to their preference for home delivery.

" At Government facilities, health workers give us a paper to buy the drug and other supplies from private pharmacies including stitching materials and gloves. Although we were informed during pregnancy follow up that delivery services are free of charges, we spent a lot of money to purchase medicine and other supplies. Those who can´t afford are discouraged by this". (FGD women participants,23 years old) .

Mothers were also concerned about the privacy they lack at the health facility. One mother described it as follows: *"I laid down on the delivery couch exposing private parts to literally everyone in the health facility. There is no privacy…different people (staffs) come frequently to the delivery room, there is no such a problem at home…". (FGD Woman participants 25 years old)*

## Discussion

The study revealed a combination of supply and demand-side factors to be responsible for the low utilization of skilled delivery services at health facilities in Afar, Ethiopia. Demand-side issues include financial constraints, distant health facilities and traditional/cultural practices. Supply-side barriers include lack of skills, poor quality services, failure to protect privacy during delivery service, lack of maternity waiting areas and limited ambulance services. Similar barriers to institutional delivery has been reported previously [13-16]. Childbirth is considered as a natural process which can be handled in a traditional way at home unless there is a problem during delivery process [17,18]. Women residing in pastoralist communities are not often accessible to health promotion and lack knowledge about the possible risks of home delivery [19]. The continued home delivery practices with no assistance from skilled birth attendants have left many lives at risk in low-income countries. Furthermore, even if mothers want to get help from skilled birth attendants, they cannot approach them easily. The health extension workers in their villages do not have the skill and facilities to assist them [20]. Besides, the number of the health extension workers assigned per kebeles is too small to cover the scattered population either through actual delivery services or health education. Increasing the number of trained health extension workers with the necessary skill to handle delivery and to refer those at risk whenever necessary could be one way of convincing the pastoralist women requiring maternal health services. The mobile lifestyle of pastoralist communities require special considerations to improve accessibility and quality of services. Addressing cultural impediments through health education, improving access through establishing maternity waiting areas, and enhancing birth preparedness through community conversation and male involvement can help prevent unnecessary sufferings and maternal and neonatal deaths [21-26]. Even though there are no costs for maternal services in public health facilities, it has been observed mothers spend money for transportation, food and purchase of drugs and supplies. These were important reasons for the low utilization of delivery services in other similar studies conducted in Ethiopia and other places as well [11,15,18,27,28]. Making sure essential supplies and drugs are available at all times and the mothers who come seeking the services return satisfied is critical to achieve higher coverage for maternity services. Lack/shortage of essential medical equipment and drugs erodes trust in health facilities and deter subsequent utilization of services either in the same or other health facilities [18,22,29]. Until the road network is expanded to ensure easy access to health facilities, local appropriate interventions to alleviate transportation problem is essential [12,21,30]. Health workers ability to effectively interact with their clients is very important in improving client's satisfaction and continued utilization of health services. Mothers in this study area reached health facilities confronting many hardships and need to be welcomed and given satisfactory treatment. Unfriendly attitude and practices including lack of privacy are known to discourage people from using health facilities even though it may be their priority [27,31]. Including health workers in the interview might have given a different opinion on the issue, and is one of the limitations of this study. This study does not also claim wider generalizability as qualitative exploration is not sufficient for generalization of findings. A quantitative study with a representative and adequate sample size based on the findings of this study would give a more comprehensive information to plan interventions that will enhance the utility of skilled birth attendants.

## Conclusion

This study identified many reasons for extremely low utilization of skilled birth attendants in Afar. In order to improve the utilization of skilled birth attendance it is important to increase awareness among women, improve birth preparedness, bringing the service closer, training service providers for quality health care, arranging waiting areas around health facilities and improving availability of supplies and ambulance services and all are critical. Further quantitative research to test and scale up culturally and contextually appropriate interventions are necessary for pastoralist population.

### What is known about this topic

- The utilization of a skilled attendant at birth has been improving in Ethiopia but with significant variations across the regional states;
- The barriers to utilization of skilled birth attendants among pastoralists communities are not fully known.

### What this study adds

- This study has identified many reasons for extreme low utilization of skilled birth attendance in the pastoral community: Women occupation, her nomadic lifestyle and lack of knowledge and awareness about skilled health services, distant health centers and inadequate transportation facilities, insufficient waiting areas in health facilities, high confidence and close proximity to traditional birth attendants were factors found to have the significant impact on low utilization of skilled birth attendance delivery.
- The study indicates the need to have a sensitive integrated and effective public health intervention on cultural and traditional practices of the pastoral community to improve utilization of skilled birth attendance health services.
